# Spermatid development in XO male mice with varying Y chromosome short-arm gene content: evidence for a Y gene controlling the initiation of sperm morphogenesis

**DOI:** 10.1530/REP-12-0158e

**Published:** 2020-04-01

**Authors:** Nadège Vernet, Shantha K Mahadevaiah, Peter J I Ellis, Dirk G de Rooij, Paul S Burgoyne

**Affiliations:** 1 Division of Stem Cell Biology and Developmental Genetics, MRC National Institute for Medical Research, The Ridgeway, Mill Hill, London NW7 1AA, UK; 2 Mammalian Molecular Genetics Group, Department of Pathology, University of Cambridge, Cambridge CB2 1QP, UK; 3 Center for Reproductive Medicine, Amsterdam Medical Center, University of Amsterdam, 1105 AZ Amsterdam, The Netherlands

The journal and the authors apologise for an error in the above titled article published in this journal (vol 144, pp 433–445 ). The authors inadvertently presented duplicate sperm images for XY and X^E^Sxr^b^O mouse testes of Fig. 6 (bottom panels). This error does not change the findings of the paper, as this figure does not give a quantitative breakdown of the proportions of different shapes.

The correct Fig. 6 is published below along with the corresponding legend.

**Figure 6:**
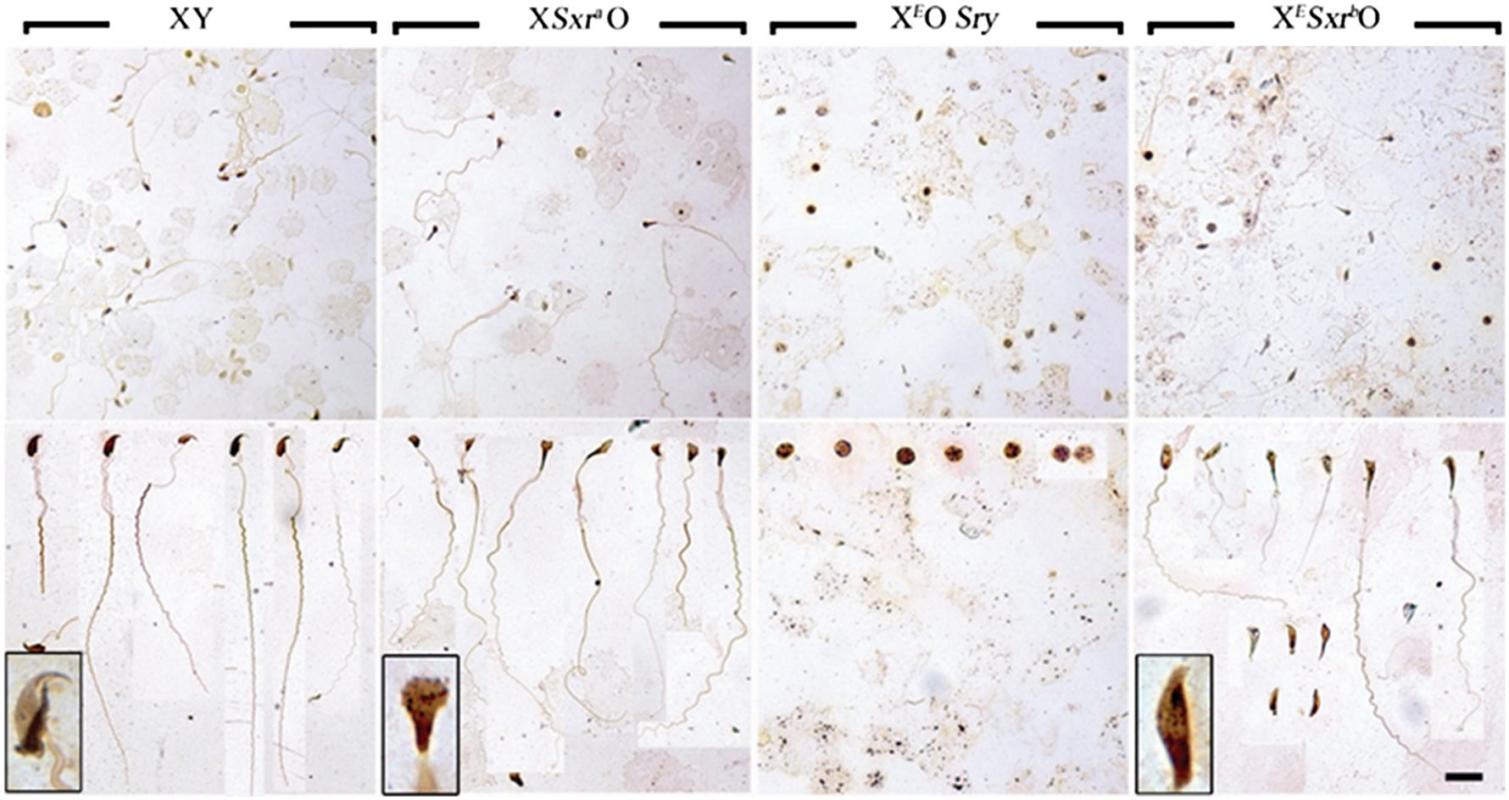
Sperm tail development is observed in X^E^Sxr^b^O male mice. Silver stained sperm smears from 6-week-old XY, XSxr^a^O, X^E^OSry and X^E^Sxr^b^O mouse testes. The top panel is a low magnification of the smear, showing the relative density of the cells found in each specimen. The bottom panel is a montage of the elongated spermatids/sperm found in each sample. Note that no elongated spermatids could be found in the X^E^OSry sample and what we believe to be the arrested round spermatids are presented instead. An example of the most characteristic sperm head for each genotype is represented at a higher magnification in the inset of the bottom panels. Scale bar: 32.5 µm (top panel); 20 µm (bottom panel).

